# Gut protozoa of wild rodents – a meta-analysis

**DOI:** 10.1017/S0031182024000556

**Published:** 2024-05

**Authors:** Simon Hunter-Barnett, Mark Viney

**Affiliations:** Department of Evolution, Ecology and Behaviour, University of Liverpool, Liverpool L69 7ZB, UK

**Keywords:** gut protozoa, meta-analysis, rodent, wild

## Abstract

Protozoa are well-known inhabitants of the mammalian gut and so of the gut microbiome. While there has been extensive study of a number of species of gut protozoa in laboratory animals, particularly rodents, the biology of the gut protozoa of wild rodents is much less well-known. Here we have systematically searched the published literature to describe the gut protozoa of wild rodents, in total finding records of 44 genera of protozoa infecting 228 rodent host species. We then undertook meta-analyses that estimated the overall prevalence of gut protozoa in wild rodents to be 24%, with significant variation in prevalence among some host species. We investigated how host traits may affect protozoa prevalence, finding that for some host lifestyles some protozoa differed in their prevalence. This synthesis of existing data on wild rodent gut protozoa provides a better understanding of the biology of these common gut inhabitants and suggests directions for their future study.

## Graphical abstract

**Figure:**
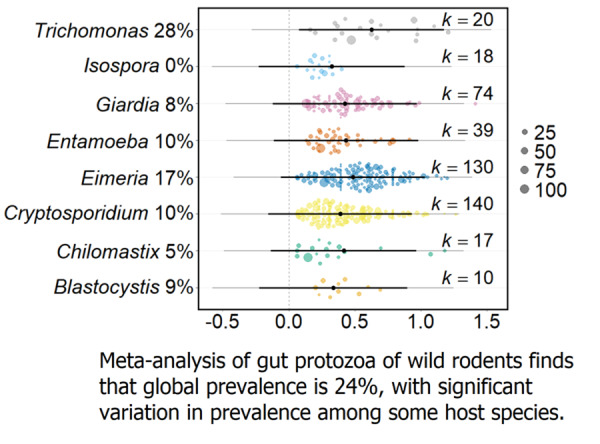

## Introduction

Protozoa are common inhabitants of the mammalian gut and an integral part of the mammalian gut microbiome (Filyk and Osborne, 2016; del Campo *et al*., 2020), but are often overlooked in host-microbiome studies in favour of prokaryotic taxa (Laforest-Lapointe and Arrieta, 2018). The protozoa of the mammalian gut can be arranged in 5 meta-groups: the Amoebozoa (e.g. *Entamoeba, Endolimax*), Apicomplexa (e.g. *Eimeria, Cryptosporidium*), Ciliophora (e.g. *Balantidium, Entodinium*), Metamonada (e.g. *Giardia, Trichomonas*), and Stramenopiles (e.g. *Blastocystis*) (Parfrey *et al*., 2011; Ruggiero *et al*., 2015; Adl *et al*., 2019; Langda *et al*., 2020; Guzzo *et al*., 2022). Gut protozoa exist across the entire parasitism – mutualism continuum, thus ranging from disease-causing parasites to long-term residents of the gut providing benefits to their host (Lukeš *et al*., 2015; Dubik *et al*., 2022), with them having both direct and indirect effects.

Mutualistic gut protozoa that provide nutritional benefits to their hosts are well-documented in ruminants (Williams *et al*., 2020; Solomon and Jami, 2021). For example, the protozoa *Eudiplodinium maggii* and *Polyplastron multivesiculatum* contribute to enzymatic degradation of plant polysaccharides in sheep (Béra-Maillet *et al*., 2005). Gut protozoa can also positively contribute to host disease resistance (Lukeš *et al*., 2015; Leung *et al*., 2018; Dubik *et al*., 2022). For example, *Tritrichomonas musculus* indirectly protects host mice against *Salmonella* infection by inducing inflammasome-driven IL-18 release (Chudnovskiy *et al*., 2016). Furthermore, *Blastocystis* subtype 4 can directly induce oxidative stress in the prokaryote *Bacteroides vulgatus*, decreasing its growth (Deng and Tan, 2022).

Negative interactions between gut protozoa and the host can result in gastrointestinal disease (Huh *et al*., 2009). Some, e.g. *Giardia* and *Cryptosporidium,* can directly cause disease by damaging and inflaming the gut epithelium (Savioli *et al*., 2006). Gut protozoa can also indirectly affect host health and disease state by changing the wider species composition of the gut microbiome (Burgess *et al*., 2017). For example, the presence of *Blastocystis* is associated with a lower abundance of beneficial prokaryotes (for example *Bifidobacterium*) whose presence normally limits infections by potential pathogens (Russell *et al*., 2011; Yason *et al*., 2019; Caudet *et al*., 2022).

Despite clear examples of parasitic and mutualistic effects of gut protozoa, it can be difficult to categorize species as either beneficial or harmful because their effects on the host can be context-dependent (Parfrey *et al*., 2011; Lukeš *et al*., 2015; Sardinha-Silva *et al*., 2022). For example, host diet, age, immune status, microbiome, and genotype, as well as protozoa genotype, can all influence the nature and strength of the interaction between a protozoan species and its host (Thompson and Monis, 2012; Ryan *et al*., 2014; Lepczyńska *et al*., 2017; Dubik *et al*., 2022). For example, *Blastocystis* can shift from being mutualistic, to becoming pathogenic when the host immune system is compromised (Scanlan *et al*., 2014).

Gut protozoa predominately have faecal – oral routes of transmission among hosts, typically through coprophagy or faecal contamination of food and / or water (Dehority, 1986; Burgess *et al*., 2017). Some gut protozoa, for example members of the Ciliophora meta-group, are dependent on the rapid faecal-oral transmission of infective stages (Michaiowski, 2005). In contrast, other species, such as *Giardia* and *Cryptosporidium,* form environmentally resistant cysts or oocysts that can persist in the environment for long periods of time allowing for more sustained transmission (Dumètre *et al*., 2012).

Host behaviour contributes to the chance of a host encountering and acquiring infective stages of protozoa (Kołodziej-Sobocińska, 2019), with more social individuals with comparatively greater social interactions having a greater chance of being exposed to protozoa (Ezenwa *et al*., 2016). For example, a meta-analysis showed that male vertebrates with a higher social status (and thus increased mating) have an overall higher parasite risk, compared to those with a lower social status (Habig *et al*., 2018). Similarly, increased parent–offspring interactions will increase the exposure of offspring to the parents’ existing protozoa community, which is seen with Ciliophora meta-group infections in ruminants (Michaiowski, 2005).

The demographics of a host population will also affect protozoa transmission in a number of ways. As host density increases this will increase the chance of protozoa transmission (Ostfeld and Mills, 2008; Ebert, 2013), but increases in host density will also affect hosts' social organization and home ranges, thus also altering individuals' risk of exposure (Bertolino *et al*., 2003; Brei and Fish, 2003; Sanchez and Hudgens, 2019). Other aspects of host biology, such as foraging behaviour, can affect transmission; for example, foraging on the ground, compared to arboreal and aerial foraging, can increase exposure to environmentally-transmitted protozoa, as is seen with *Entamoeba* in baboons and *Isospora* in birds (Dolnik *et al*., 2010; Barelli *et al*., 2020).

An individual's diet, immune state, and pre-existing microbiome (both prokaryotic and eukaryotic) can also influence the chance of a protozoan successfully establishing in the gut (Thursby and Juge, 2017; Kołodziej-Sobocińska, 2019; Coyte *et al*., 2021). Host diet can alter nutrient availability, allowing the establishment and maintenance of different gut protozoan communities (Zhang *et al*., 2022). For example, the relative abundance of *Entodinium* in sheep rumen fluid changes in response to different diets (Henderson *et al*., 2015; Zhang *et al*., 2022). Host immune state can affect the initial establishment and subsequent persistence of protozoa in the gut (Evering and Weiss, 2006; Sardinha-Silva *et al*., 2022). Long-term co-evolution of protozoa with their hosts has allowed many protozoa to evolve to be either tolerated by and / or evade the host immune response (Zambrano-Villa *et al*., 2002; Macpherson *et al*., 2005; Schmid-Hempel, 2009; Tanoue *et al*., 2010; Sardinha-Silva *et al*., 2022). A host's pre-existing microbiome can also affect subsequent establishment of other taxa (Coyte *et al*., 2021). For example, some Ciliophora species in the livestock rumen microbiome require a pre-established prokaryotic community for their survival (Michaiowski, 2005). Furthermore, there is often an obligate pattern of succession in establishment; for example, in many ruminants *Entodinia* spp. is the primary colonizer after which other Ciliophora species establish (Michaiowski, 2005). Competition among microbial species for nutrients and other resources results in the generation of niches within the gut, controlling the diversity of protozoa that can establish (Pereira and Berry, 2017). For example, *Tritrichomonas musculus* competes with prokaryotic taxa for dietary fibre, a resource essential for *T. musculus* colonization (Wei *et al*., 2020). Prokaryotic taxa can produce molecules that limit the establishment of protozoa; for example, *Lactobacillus reuteri* and *L. acidophilus-*derived factors can inactivate *Cryptosporidium* oocysts (Foster *et al*., 2003).

Most of what is known about gut protozoa of mammals comes from studies of people, livestock, and laboratory animals. In contrast, there are limited studies describing the diversity of gut protozoa in wild mammals, and what drives variation in protozoa composition. The gut microbiomes of laboratory and domesticated animals are likely to be quite distinct from those of their wild counterparts (Prabhu *et al*., 2020; Bowerman *et al*., 2021), so there is a need to study wild animals in greater detail. The Rodentia are a highly speciose order of mammals (Fabre *et al*., 2012), but their gut protozoa are not well described. As with most mammals, the majority of described gut protozoa in wild rodents are parasitic, rather than mutualist (Parfrey *et al*., 2014). In part, this may be because there has been a focus on parasitic protozoa of rodents, given their potential as sources of zoonotic infection (Meerburg *et al*., 2009; Han *et al*., 2015). There has been limited effort to describe the mutualistic gut protozoa of wild rodents, except in those species with comparatively enhanced digestive efficiency, e.g. the capybara, *Hydrochoerus hydrochaeris* (Borges *et al*., 1996).

To further our understanding of mammalian gut protozoa we have systematically reviewed records of protozoa present in the gut microbiome of wild rodents. This, as far as we are aware, has not been done before. After describing the protozoa known to infect the gut of wild rodents, we then sought to understand how the prevalence of their infection varies among different protozoa and among different hosts, and how aspects of host biology affect this.

## Materials and methods

### Literature search

**Figure 1.**
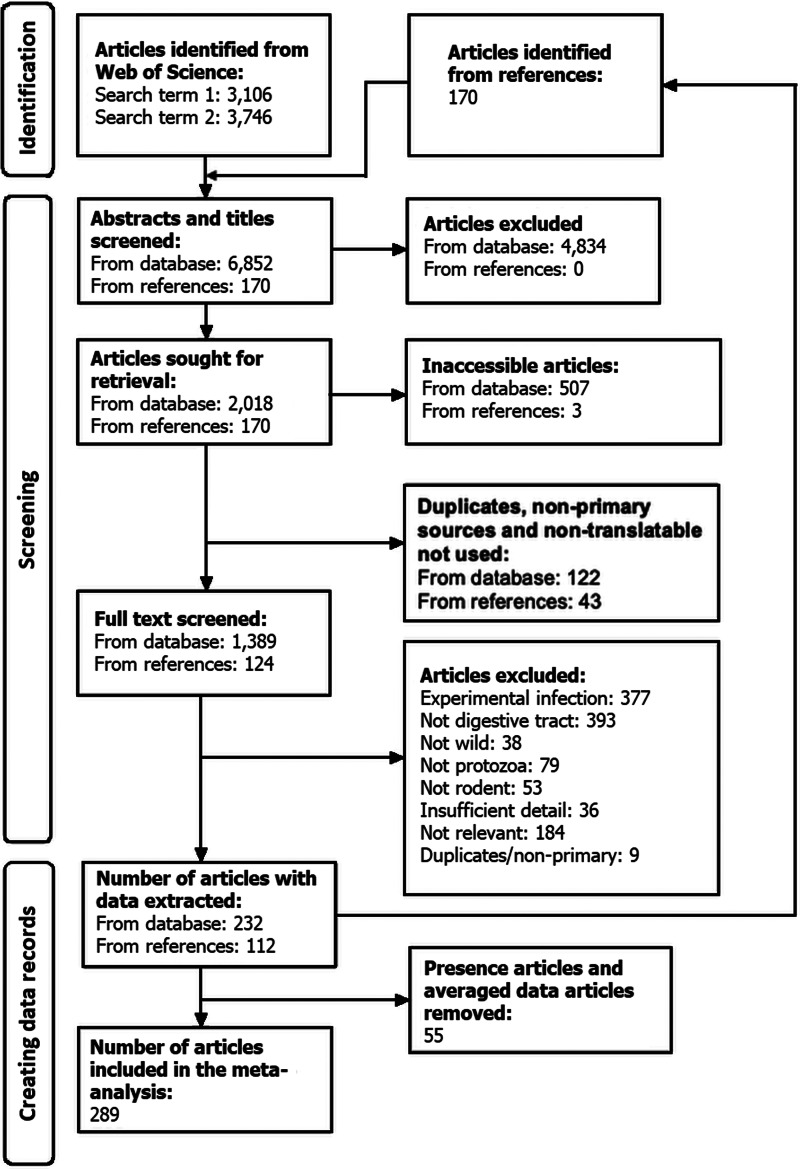
PRISMA diagram showing the source of articles and the subsequent screening stages used to generate the data records used in the meta-analysis.

We searched the Web of Science for articles describing gut protozoa infections of wild rodents, following PRISMA guidelines (Moher *et al*., 2009; Page *et al*., 2021). We used 2 independent searches: the first in March 2020, using the 4 search terms ‘infection rodent protozoa gut’, ‘gut protozoa rodent’, ‘parasite rodent gut’ and ‘eukaryotic microbiome rodent’, where each term was searched for simultaneously in ‘Topic’; the second in April 2020, performed as above but using the search term ‘protozoa wild rodent’, with an additional 7 search terms (wild-type, ‘wild type’, model, and the 4 search terms used in March 2020) using the ‘NOT’ command. This second search was used to avoid articles reporting studies on laboratory rodents while excluding any potential duplicate articles from the first search. In all, this resulted in retrieving 6852 articles, which were then screened and reduced to 2018 articles that were carried forward for full-text screening (Fig. 1), where we retained articles that reported naturally occurring protozoa infections of the gut of a wild rodent. We excluded articles that did not give the location of the wild rodent, as too those that did not identify the rodent host or the protozoan parasite to the genus level. Once data were extracted, their reference lists were searched to identify any additional potential articles not identified in the literature search; this identified a further 112 articles, from which data were also extracted.

### Data extraction

We categorized articles as either (i) a report of the presence of a protozoan (henceforth ‘presence’ article) or (ii) a report of the protozoan prevalence (henceforth ‘prevalence’ article). We created data records by extracting the following data from articles: host species, protozoa species, geographical location (as continent, country, and latitude and longitude (if provided)), diagnostic technique and year sampled. A single article could produce multiple data records. We recorded protozoa prevalence from prevalence articles, where necessary calculating this from reported data. We used median prevalence when prevalence ranges were reported; we used mean prevalence when different prevalence values were reported for host sub-species and species complexes; if multiple prevalence values were reported for con-generic protozoan species, a mean protozoa genus prevalence was calculated. Weighted means were calculated based on the sample size of the individual reports. For articles that used multiple diagnostic techniques for the same rodents we recorded either (i) the combined prevalence from the multiple diagnostic techniques reported in the article or (ii) if the combined prevalence was not given, then we calculated the average prevalence of the multiple diagnostic techniques, and then reported the diagnostic technique for these records as ‘Mixed’.

From these data we generated a meta-table recording the presence of different protozoa in the gut of wild rodents, with data recorded at the genus level for protozoa, and at species level for the host. Rodent host taxonomy was after the Handbook of the Mammals of the World (Wilson *et al*., 2017). Protozoa genera were assigned to 1 of 5 meta-groups: Amoebozoa; Apicomplexa; Ciliophora; Metamonada and Other (Adl *et al*., 2019). A generalized linear model (GLM) with a Poisson error distribution (Zuur and Ieno, 2016) was used to determine if the number of protozoa genera identified in a rodent species was dependent on the surveying effort (i.e. the number of records) for that rodent species.

### Analysis of protozoa prevalence

Our aim was to explore the causes of variation in protozoa prevalence in the gut of wild rodents. The records for which an average prevalence was calculated were removed, but the average prevalence record was kept (Fig. 1). This was to ensure that there was no pseudo-replication of the data. Each data record was assigned an article ID and a unique record number (URN). We used the metafor package within RStudio to conduct all meta-analyses (v2.4.0, Viechtbauer, 2010). Our general strategy was: (i) create a base restricted maximum likelihood estimator (REML) model with only random effects that would be used throughout the following data analyses, (ii) investigate if there was variation in the prevalence of protozoa across different rodent host species, (iii) identify variables contributing to variation in protozoa prevalence, and (iv) investigate any potential publication and methodological biases in the dataset.

The base REML model listed article ID, URN, diagnostic technique and host phylogeny as random factors. Host phylogeny accounted for potential variation in prevalence due to hosts' shared evolutionary history (Koricheva *et al*., 2013). The phylogeny was created using the Open Tree of Life (OTL) database (Hinchliff *et al*., 2015) and the rotl R package (v3.0.14, Michonneau *et al*., 2016). Some species were not present in the OTL and so these were manually added to the tree. Grafen's method was used to compute branch lengths using the ape R package (Grafen, 1989; Paradis *et al*., 2004). The final phylogenetic tree is available in Supplementary Figure 1. Diagnostic technique was included as a random factor to account for potential variation in prevalence due to the diagnostic technique used. In all models, the dependent variable was double-arcsine transformed prevalence (Wang, 2023), with this transformation fitting the assumptions of normality required for meta-analyses (Barendregt *et al*., 2013). Recent work has recommended not using double-arcsine transformation in meta-analyses (Lin and Xu, 2020; Röver and Friede, 2022), and so we completed all analyses on both double-arcsine and single-arcsine transformed data, finding that for all models the results and conclusions drawn were identical (Hunter-Barnett, 2023). To test whether various factors significantly affect protozoan prevalence we added these factors as a fixed effect (henceforth called a moderator) to the base model.

We used the rma.mv function in the base model to calculate the overall double-arcsine transformed prevalence, with this result back-transformed to obtain the summary percentage prevalence and 95% confidence intervals (CI) (Wang, 2023). The number of records included in the model (k) was also recorded. Heterogeneity of prevalence was examined using the *I*^2^ statistic, which is the proportion of variance in effect sizes that is not attributable to sampling (i.e. error) variance (Higgins *et al*., 2003). The proportion of *I*^2^ attributable to differences in article ID, URN, diagnostic technique, and host phylogeny was calculated using the i2_ml function in the orchaRrd R package (Nakagawa *et al*., 2021).

To investigate how gut protozoa prevalence varied among different host taxa we performed 2 meta-regressions of gut prevalence, incorporating host family or host species as the moderator. The moderator ‘protozoa genus’ and the subsequent interaction terms with the host family and host species were also included in the models, but only incorporating either where there were at least 10 records, thus guarding against bias caused by small sample sizes (Lin, 2018). Significant moderators indicated that they affected mean protozoa prevalence; significance was defined by examining the *Q*_M_ statistic and marginal *R*^2^ values were calculated to establish how much heterogeneity in prevalence was described by the moderators, using the r2_ml function in the orchaRd R package (Nakagawa and Schielzeth, 2013; Nakagawa *et al*., 2021).

When we found significant effects of interactions between protozoa and host, we examined these further by dividing the host family or host species into subgroups and running separate meta-regressions for each subgroup, with protozoa genus as the moderator. Only the host subgroups that had at least 2 protozoa genera, with at least 10 records per protozoa genus, were tested. If there was a significant effect of protozoa genus we conducted pairwise comparisons between protozoa genera, using Tukey *post hoc* comparisons, which was done by re-running the meta-regression and excluding the intercept, and using the multcomp R package to compare combinations of protozoa genera (Hothorn *et al*., 2008). We used the holm method to correct for multiple testing (Holm, 1979). Finally, the average double-arcsine transformed prevalence for each subgroup within each moderator was obtained by using the subset function within the rma.mv model. Orchard plots (including 95% CIs and 95% prediction intervals) were used to show differences in prevalence among subgroups (Nakagawa *et al*., 2021). Prediction intervals represent the range of prevalence in which the prevalence of a new observation would fall (IntHout *et al*., 2016). Precision, as the inverse of the standard error for each record, was used in these plots, where a larger precision equates to a larger sample size.

To investigate if geographical differences were contributing to variation in protozoa prevalence, 3 geographical moderators were included: longitude, latitude and continent. Latitude and longitude were converted from degrees, minutes and seconds format to the decimal degrees format using OSMscale (v0.5.1, Boessenkool, 2017), so generating a continuous variable. In this model, the interactions of latitude and longitude with continent were also included as moderators. Additionally, protozoa genus and its interactions with each of the 3 geographical moderators were also included, to account for variation stemming from different protozoa genera.

To investigate if host behaviour was contributing to variation in protozoa prevalence, host behaviour moderators were created for each host species. A single resource was used to extract behavioural information (Wilson *et al*., 2017), forming eight moderators that we hypothesized may affect interactions between rodent hosts, so affecting protozoa transmission (Ostfeld and Mills, 2008; Sarkar *et al*., 2020). The 8 moderators were: (i) host density, (ii) host home range (i–ii extracted as quantitative values), (iii) host dispersal distance (and then made into <1 and >1 km categories), (iv) typical social grouping (solitary or group-living), (v) typical mating system (monogamous or polygamous), (vi) development type (altricial or precocial) (iv–vi recorded as categorical data), (vii) social system (with 11 sub-groups), and (viii) typical lifestyle (general behaviour, locomotion and morphology) (Derrickson, 1992; Wilson *et al*., 2017). If behavioural information was not available for a species, family characteristics were used but only if this characteristic applied to all species in that family. These 8 moderators were tested separately in a meta-regression, each with protozoa genus included and the relevant interaction term.

To investigate if diagnostic technique affected reported gut protozoan prevalence, diagnostic technique was added as a moderator in a meta-regression. This model removed diagnostic technique from the random effects. *Post hoc* tests were completed as described above. A second meta-regression was conducted, with precision as a moderator, to determine if sample size affected protozoa prevalence. A funnel plot was used to visualize publication bias, with an asymmetrical plot indicating missing effect sizes, potentially from publication bias (Koricheva *et al*., 2013; Shi and Lin, 2019). A trim-and-fill test (Duval and Tweedie, 2000) was used to detect missing effect sizes and predict the average effect size if these were to be included in the analysis.

## Results

### Protozoa and host records

A total of 344 suitable articles were identified from the literature search, published between 1915 and 2020 (Supplementary Table 1). From these, 2245 data records of 44 genera of protozoa, across 69 countries (Supplementary Table 2), encompassing all 5 protozoa meta-groups (Amoebozoa 95 records, 4 genera; Apicomplexa 1725, 12; Ciliophora 38, 14; Metamonada 368, 11; Other 19, 2 (*Blastocystis* and *Pharyngomonas*)), were recorded in the gut of wild rodents. The most data records were of Apicomplexa and Metamonada protozoa, and the most common protozoan genera for which there were data records were *Cryptosporidium, Eimeria* and *Giardia*. 275 rodent host species were identified from 110 genera and 21 families, with large variation in the number of data records generated for each host species, with the most common data records for *Apodemus, Microtus* and *Rattus*.

From the 2245 data records, there were 1886 records of gut protozoa in wild rodents. Of the 275 host species, 228 had a confirmed protozoan in the gut (combining both presence and prevalence articles) (Table 1; Supplementary Table 3). In total 44 genera of protozoa are present in the gut of wild rodents, though genera were highly variable in the number of host species from which they have been reported. Only 7 protozoa genera (*Chilomastix, Cryptosporidium*, *Eimeria, Entamoeba*, *Giardia, Isospora*, *Trichomonas*, from Apicomplexa, Metamonada and Amoebozoa) were recorded in the gut of more than 10 host species. *Eimeria* was recorded as the most widely host-distributed distributed protozoa genus, identified in 194 (85% of 228) host species. In comparison, 27 protozoa were reported from only one host species, including 13 (of 14) Ciliophoran genera.

**Table 1.** Protozoa found in the gut of wild rodents

Rodent Family	Amoebozoa				Apicomplexa												
	*Acanthamoeba (1)*	*Amoeba (1)*	*Endolimax (5)*	*Entamoeba (29)*	*Adelina (1)*	*Caryospora (1)*	*Cryptosporidium (59)*	*Cyclospora (1)*	*Cystoisospora (1)*	*Dorisiella (1)*	*Eimeria (194)*	*Isospora (22)*	*Klossia (1)*	*Sarcocystis (1)*	*Toxoplasma (1)*	*Tyzzeria (2)*	*Monocystis (1)*
Aplodontiidae																	
Bathyergidae																	
Calomyscidae																	
Castoridae																	
Caviidae																	
Chinchillidae																	
Cricetidae																	
Ctenomyidae																	
Dasyproctidae																	
Echimyidae																	
Erethizontidae																	
Geomyidae																	
Gliridae																	
Heterocephalidae																	
Heteromyidae																	
Muridae																	
Nesomyidae																	
Sciuridae																	
Spalacidae																	
Thryonomyidae																	
Zapodidae																	

Rodent Family	Cilio	Metamonada			Other										
	*Balantidium (2)*	Other (1)	*Giardia (50)*	*Hexamita (4)*	*Octomitus (3)*	*Spironucleus (3)*	*Hexamastix (2)*	*Chilomastix (13)*	*Retortamonas (4)*	*Tetratrichomonas (1)*	*Trichomonas (21)*	*Dientamoeba (1)*	*Tritrichomonas (3)*	*Blastocystis (8)*	*Pharyngomonas (1)*
Aplodontiidae															
Bathyergidae															
Calomyscidae															
Castoridae															
Caviidae															
Chinchillidae															
Cricetidae															
Ctenomyidae															
Dasyproctidae															
Echimyidae															
Erethizontidae															
Geomyidae															
Gliridae															
Heterocephalidae															
Heteromyidae															
Muridae															
Nesomyidae															
Sciuridae															
Spalacidae															
Thryonomyidae															
Zapodidae															

Protozoa are grouped by meta-group, and then alphabetically, with the number in parentheses showing the number of host species from which that protozoa had been identified. ‘Cilio’ are the ciliophora mega-group. Rodent taxa are shown by rodent families; the same data for rodent species are shown in Supplementary Table 3.

The number of protozoa genera identified in the gut of each wild rodent host species was highly variable. Nineteen host species had 5 or more protozoa, with most of these belonging to the Muridae and Cricetidae. The greater capybara (*H. hydrochaeris*) had the most (17), followed by the brown rat (13, *Rattus norvegicus*) and the black rat (11, *R. rattus*). Most (145, 64% of 228) rodent species had just a single protozoan recorded, and these host species were from 14 rodent families. The number of different protozoa identified in rodent species was linked to how intensively that host species was surveyed; specifically, there was a significant, positive relationship between the number of data records for a rodent host and the number of different protozoa identified (GLM: *F*_1,226_ = 145.5, *P* < 0.001).

### Protozoa prevalence

A total of 1237 (of 2245) data records (after the removal of pseduoreplicated data records and presence records) were used to investigate variation in the prevalence of protozoa in the wild rodent gut. A total of 255 rodent species were surveyed across 289 articles, from 102 host genera and 21 host families, and 36 protozoa genera were used in the meta-analysis.

Across all wild rodents, the average prevalence of gut protozoa infection was predicted to be 23.7% (95% CI 4.8–48.5, *k* = 1237). However, the trim-and-fill test detected asymmetry in the funnel plot, with 187 missing effect sizes being added above the mean. Adding these 187 effect sizes adjusted the overall protozoa prevalence to 32.9% (CI 30.6–35.1, *k* = 1424). There was no change in prevalence over the time period of the records (*Q*_M_ = 0.023, *P* = 0.880, *k* = 1015).

There was substantial variation in the prevalence of protozoa infection in the dataset (*I*^2^ = 97.8%), with much of this variation stemming from differences among individual data records (32.3%) and differences attributed to the article ID of the data record (32.0%). However, host phylogeny explained 26.9% of the variation in protozoa prevalence, and diagnostic techniques 6.5%.

**Figure 2.**
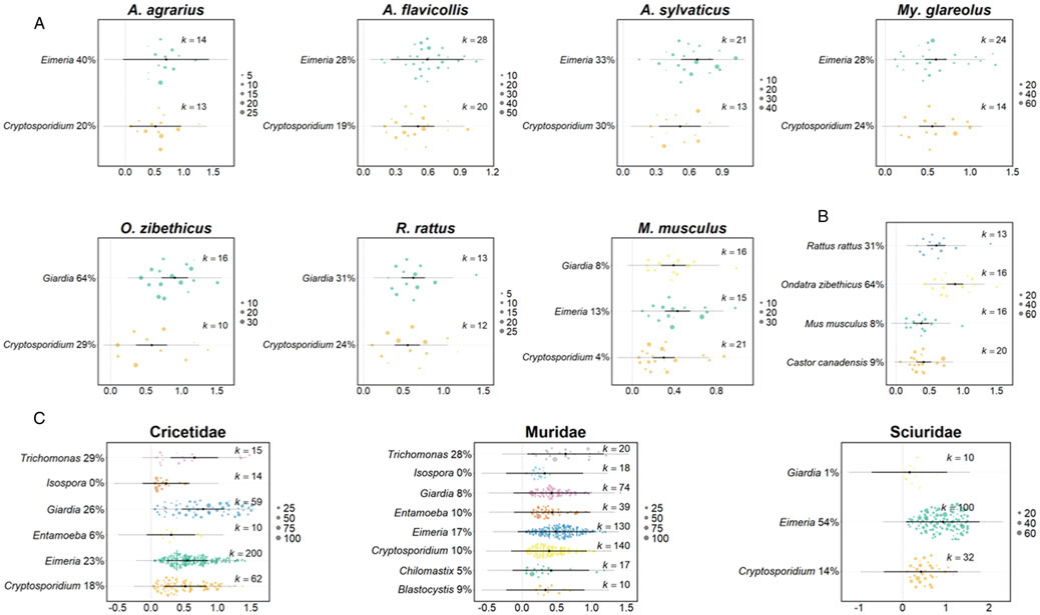
The prevalence of (A) protozoa in 7 host species, (B) *Giardia* in 4 host species, and (C) protozoa in the rodent families Cricetidae, Muridae and Sciuridae. In all, prevalence, shown on the x-axis, is double-arcsine transformed; the x-axis differs among panels. The black point indicates the estimated average prevalence, with the bold lines showing 95% CIs, and thin lines showing 95% prediction intervals. The size of the points are scaled to precision (shown on the scale on the right-hand side of each panel), and *k* indicates the number of records for that protozoan. The back-transformed predicted prevalence percentage is provided next to the protozoa genus label.

Host species differed significantly in their prevalence of gut protozoa (host species moderator *Q*_M_ = 41.7, *P* < 0.001; interaction protozoan genus *Q*_M_ = 122.4, *P* < 0.001, *k* = 538; Figure 2A; Supplementary Table 4). We examined 7 host species (*Apodemus agrarius, A. flavicollis, A. sylvaticus, Mus musculus, Myodes glareolus, Ondatra zibethicus* and *R. rattus*) more closley, which showed that protozoan genus was only a significant moderator of prevalence for the muskrat (*O. zibethicus*), such that it had a higher prevalence of *Giardia* (64.2%) compared with *Cryptosporidium* prevalence (29.2%); for the other 6 host species there was no effect of protozoan genus on prevalence. The prevalence of *Giardia* in the muskrat was significantly higher compared to hosts *Castor canadensis, M. musculus* and *R. rattus* (*Q*_M_ = 18.8, *P* < 0.001, *k* = 65, Fig. 2B).

There was no significant difference in the predicted prevalence of protozoa infection among different rodent families, though there were significant differences in interactions between protozoa genus and host family (host family moderator *Q*_M_ = 1.5, *P* = 0.59, interaction protozoan genus *Q*_M_ = 107.6, *P* < 0.001, *k* = 1111); thus, host families had different prevalence of gut protozoa infection for certain genera of protozoa. We investigated this further by analysing different rodent families separately, finding that 3 host families – Cricetidae, Muridae and Sciuridae – had at least 2 protozoa genera, with at least 10 records per protozoa genera, and protozoa genus was a significant moderator of prevalence in all (Fig. 2C; *Q*_M_ = 33.2, *P* < 0.001, *k* = 448, *Q*_M_ = 46.2 *P* < 0.001, *k* = 360, *Q*_M_ = 42.0, *P* < 0.001, *k* = 142 for Cricetidae, Muridae and Sciuridae, respectively).

### Factors affecting prevalence of infection

**Figure 3.**
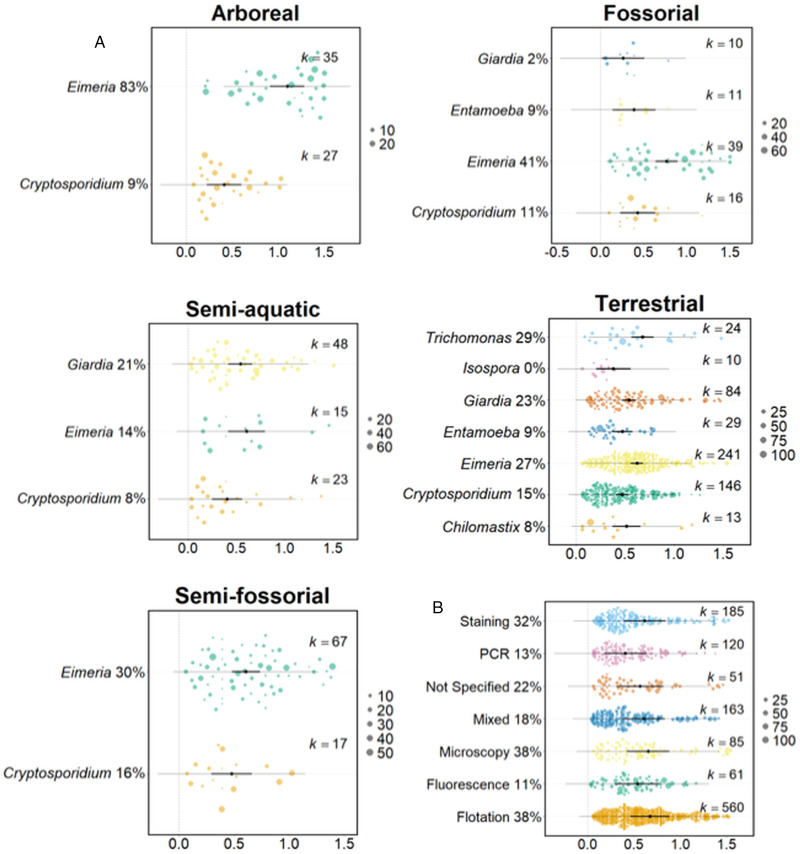
The average prevalence of protozoa (A) across 5 different host lifestyles and (B) according to method of diagnosis. In all, prevalence, shown on the x-axis, is double-arcsine transformed; the x-axis differs among panels. The black point indicates the estimated average prevalence, with the bold lines showing 95% CIs and thin lines showing the 95% prediction intervals. The size of the points are scaled to precision (shown on the scale on the right-hand side of each panel) and *k* indicates the number of records for the specified protozoa or diagnostic method. The back-transformed predicted prevalence percentage is provided next to the protozoa genus name or diagnostic method. In B, the *p* values for *post hoc* comparisons between the following diagnostic techniques with significant differences are: PCR: flotation <0.001; PCR: microscopy 0.017; PCR: mixed 0.038; PCR: staining 0.024.

Variation in host lifestyle – arboreal, fossorial, semi-aquatic, semi-fossorial and terrestrial – did not significantly affect protozoa prevalence. However, there was a significant interaction between host lifestyle and protozoan genus (lifestyle moderator *Q*_M_ = 1.06, *P* = 0.983, interaction protozoan genus *Q*_M_ = 57.3, *P* = 0.003, *k* = 988). We examined this further, finding that for arboreal, fossorial and terrestrial host lifestyles, protozoa genus had a significant effect on prevalence (*Q*_M_ = 33.8, *P* < 0.001, *k* = 62, *Q*_M_ = 15.9, *P* = 0.001, *k* = 76, *Q*_M_ = 26.3, *P* < 0.001, *k* = 547 for arboreal, fossorial and terrestrial lifestyles, respectively). Specifically, *Eimeria* had a significantly higher prevalence in the gut of arboreal and fossorial rodents (82.9% and 40.8%) compared with other protozoa (Fig. 3A). *Eimeria* was also significantly more prevalent in terrestrial rodents compared to *Cryptosporidium* (26.8% and 15.0%, respectively); *Trichomonas* was significantly more prevalent in terrestrial rodents (28.5%), compared to *Entamoeba* (8.9%) and *Cryptosporidium* (15.0%). Different protozoa genera did not have a significantly different prevalence in either semi-aquatic or semi-fossorial rodents.

There was no evidence that geographical location nor rodent host sociality as measured by 7 variables (home range size; dispersal distance; density; social system; binary social system; development type; and mating system) affected protozoa prevalence.

### Methodological effects

The use of eight different diagnostic techniques were recorded from the articles. The most common were flotation (550 records), staining (185) and PCR (120). There was significant variation in protozoa prevalence according to the diagnostic technique used (*Q*_M_ = 23.62, *P* < 0.001, *k* = 1,225, Fig. 3B). *Post hoc* comparisons showed that PCR-based diagnoses found a significantly lower prevalence of protozoa (13.2%) compared to microscopy, flotation and staining methods (38.3%, 37.5% and 32.4% respectively). Using multiple diagnostic techniques did not increase the report of protozoa prevalence compared with using any single diagnostic method, except PCR.

A meta-regression did not detect a significant relationship between study precision and protozoa prevalence (*Q*_M_ = 0.920, *P* = 0.338, *k* = 1,237), indicating that across the whole dataset, larger sample sizes did not reveal a higher prevalence of protozoa.

## Discussion

This work found that 44 genera of protozoa from all 5 mega-groups have been recorded from the gut of wild rodents. Some genera – *Cryptosporidium, Eimeria, Entamoeba, Giardia* – occurred commonly, in 29 rodent host species, consistent with their wide host range among vertebrates more generally (Appelbee *et al*., 2005; Ryan *et al*., 2014; Duszynski, 2021; Zanetti *et al*., 2021). *Isospora* also had a wide rodent host range, being recorded from 22 species, contrasting with previous suggestions that rodents are not its natural hosts (Trefancová *et al*., 2019). However, *Blastocystis* was found in only 8 rodent species, therefore contrasting with reports of its wide host range (Alfellani *et al*., 2013). Other protozoa appear to have a much more narrow host range: *Balantidium* was found in only 2 rodent host species, consistent with them acting as potential carriers while its infection predominates in pigs and primates (Schuster and Ramirez-Avila, 2008). Many studies of wild rodents have likely focussed on protozoa that are parasites, and so there may be an under representation of mutualistic species of protozoa.

These records of infection require accurate identification of the protozoan taxa, which is not always straightforward, and can be further complicated by changes to taxonomic names and reclassification. For example, *Trichomonas* was reported from 21 rodent species, despite being commonly associated with the digestive tract of birds and the human vagina (Malik *et al*., 2011), suggesting that overall it has a wide vertebrate host range. However, some *Trichomonas* spp. are synonymous with *Tritrichomonas* spp. (Burr *et al*., 2012), with *Tritrichomonas* being described from the laboratory rodent gut microbiome (Escalante *et al*., 2016), but was only reported in one wild rodent species in the present study. Combining the presence records of the synonymous *Trichomonas* and *Tritrichomonas* spp. then shows that it has a wider rodent host range. Similarly, the protozoa *Spironucleus muris* is known to colonize the gut of many laboratory rodents (Jackson *et al*., 2013) but was only reported from 3 wild rodent species. However, *Spironucleus* spp. are often misidentified as *Hexamita* spp. and reclassifications are common (Jørgensen and Sterud, 2007; Jackson *et al*., 2013). *Hexamita,* is better known for infecting fish and birds (Uldal and Buchmann, 1996; Cooper *et al*., 2004), but has records in 4 rodent species. Combining *Spironueclus* and *Hexamita* presence records leads to the conclusion that it has a wider rodent host range. Clarifying and stabilizing protozoa taxonomy would help improve our understanding of the host range of gut protozoa of wild rodents.

Three protozoa genera – *Adelina, Klossia, Monocystis* – reported from wild rodents in the present study are also known to infect arthropods and earthworms (Field and Michiels, 2005; Bekircan and Tosun, 2021; Zeldenrust and Barta, 2021). While these rodent records could be true infections of rodents, it is also possible that these records are actually because rodents ate invertebrates harbouring these protozoa. Furthermore, *Acanathomoeba* spp. and *Amoeba* spp. are typically considered to be free-living (Rodríguez-Zaragoza, 1994) but were each identified from one rodent species, and these putative rodent infections are more likely transient infections. Similarly, the genus *Pharyngomonas* (originally *Trichomastix*) was recorded in the naked mole rat, *Heterocephalus glaber,* though it is a halophilic protozoan (Park and Simpson, 2015) and so it unlikely to be a natural resident of this rodent.

Meta-analysis of these data found that the global protozoa prevalence of wild rodents is 23.7%, which is slightly higher than previous estimates for individual protozoa genera in wild rodents e.g. 18%, 19.8% and 20.1% for *Blastocysti*s, *Cryptosporidium* and *Giardia,* respectively (Li *et al*., 2017; Zhang *et al*., 2021; Barati *et al*., 2022). It is important to note that this global estimate may be conservative since many studies included in this meta-analysis sought particular protozoa taxa, rather than any protozoa taxa.

We found that rodent host species differed significantly in the prevalence of protozoa infection, but that protozoa genera did not differ in their prevalence within a host species. This, combined with no evidence of geographical effects on protozoa prevalence, suggests that the rodent species-level effect on prevalence applies widely to different protozoa, perhaps driven by host species-specific traits or wider demographic effects. The exception to this finding was the muskrat, *Ondatra zibethicus*, where *Giardia* had a significantly higher prevalence than *Cryptosporidium. Giardia* cysts are detected in water more frequently than *Cryptosporidium*, which may explain the higher *Giardia* prevalence in the semi-aquatic muskrat (Cacciò *et al*., 2005; Ganoe *et al*., 2020). There were no differences in protozoa prevalence among different rodent families. For some rodent families – Cricetidae, Muridae, Sciuridae – there were protozoa-level effects, which warrants further investigation into the underlying cause and mechanism.

The meta-analysis found no effect of host sociality on protozoa prevalence, which is interesting given that there are rodent species-level effects and an increasing awareness of the importance of social interactions affecting transmission of gut microbes (Grieneisen *et al*., 2017; Raulo *et al*., 2021). However, other work focussed on parasitic taxa has shown that there is no relationship between rodent sociality and endoparasite load (e.g. Bordes *et al*., 2007; Hillegass *et al*., 2008). Our analyses also found no evidence for an effect of host population density or home range size on protozoa prevalence, despite evidence that both are associated with the chance of incidental transmission of gut microbes in wild mammals (Li *et al*., 2016; Sarkar *et al*., 2020; Wikberg *et al*., 2020). Together, this suggests that other rodent species-level traits not considered here are important in affecting the prevalence of protozoa infection. These data do not include any information on hosts' immune responses or immune state, and this could affect the amount of detectable infection in host species.

Our analyses also found no effect of host lifestyle on protozoa prevalence, which contrasts with previous suggestions that arboreal and semi-arboreal lifestyles disfavour faecal-oral protozoa transmission, potentially leading to a comparatively lower protozoa prevalence in animals with such lifestyles (Gilbert, 1997; Barelli *et al*., 2020). However, our analyses did find that for arboreal, fossorial, and terrestrial lifestyles there were protozoa-level effects. Specifically, *Eimeria* was comparatively more prevalent in arboreal and fossorial rodents; *Trichomonas* and *Eimeria* were comparatively more prevalent in terrestrial rodents. However, it is important to note that these findings may be driven by protozoa-level effects within the Sciuridae, Muridae and Cricetidae. Specifically, (i) *Eimeria* was comparatively more prevalent in the Sciuridae, and many Sciuridae species were classed as either arboreal or fossorial and (ii) *Trichomonas* and *Eimeria* were comparatively more prevalent in the Muridae and Cricetidae and many Muridae and Cricetidae species were classed as terrestrial rodents. Thus, it is probable that the protozoa-levels effects seen within the arboreal, fossorial and terrestrial rodents may be confounded by rodent family-level taxonomic effects. Furthermore, the meta-analysis did not include data on other environmental factors known to impact transmission of gut microbes in wild mammals, such as habitat type and seasonality (Kołodziej-Sobocińska, 2019; Barelli *et al*., 2020). Thus, the impact of these traits on transmission, and therefore protozoa prevalence, were not addressed in this meta-analysis.

Concerning diagnosis of infection, we found that PCR reported comparatively lower prevalence of infection. This result is perhaps unexpected because PCR is typically highly sensitive (McHardy *et al*., 2014; Compton, 2020). However, this PCR effect may be due to difficulties in extracting DNA from protozoa (oo)cysts, whereas (oo)cysts are often readily detected (and diagnosed) by microscopical examination (Hawash, 2014). Furthermore, the taxonomic tight-specificity of PCR diagnosis contrasts with the other diagnostic methods that can detect a broader range of taxa (den Hartog *et al*., 2013; Compton, 2020). In the future metagenomic sequencing may be beneficial to get a more broad-based measure of the protozoa community in animal guts.

Publication bias was detected in the dataset, driven by a lack of studies reporting high prevalence of infection. Publication bias normally arises from a tendency to not publish studies with less significant results and / or smaller sample sizes (Shi and Lin, 2019); instead, one may expect publication bias in favour of reporting high protozoa prevalence. Therefore, the comparative rarity of reports of high prevalence suggests that high protozoa prevalence is actually rare. Our meta-analysis has also highlighted how taxonomic reclassifications and revisions of protozoa make it hard to define, even at the genus level, which protozoa can colonize the rodent gut.

In summary, this analysis is the first, of which we are aware, synthesizing information about the gut protozoa of wild rodents, estimating the global prevalence of gut protozoa, and identifying host species-level effects on protozoa prevalence. To investigate these patterns further new studies will be required that, for example, generate data on individual- and population-level traits of hosts to understand the context-specific role of host behaviour on protozoa infection. Given the current focus on parasitic gut protozoa, future studies should also seek to include putative mutualistic protozoa, so furthering our understanding of the gut eukaryome of wild rodents.

## Supporting information

Hunter-Barnett and Viney supplementary material 1 Hunter-Barnett and Viney supplementary material

Hunter-Barnett and Viney supplementary material 2 Hunter-Barnett and Viney supplementary material

Hunter-Barnett and Viney supplementary material 3 Hunter-Barnett and Viney supplementary material

Hunter-Barnett and Viney supplementary material 4 Hunter-Barnett and Viney supplementary material

Hunter-Barnett and Viney supplementary material 5 Hunter-Barnett and Viney supplementary material

## Data Availability

The data we have analysed are available from within our manuscript; the source data are in the papers included in our meta-analysis.

## Supplementary material

The supplementary material for this article can be found at https://doi.org/10.1017/S0031182024000556.
