# Supplementary figures and images for: Gut protozoa of wild rodents – a meta-analysis
Source: Parasitology. 2024 May 8;151(6):594–605. doi: 10.1017/S0031182024000556 (PMC11427965; doi:10.1017/S0031182024000556)

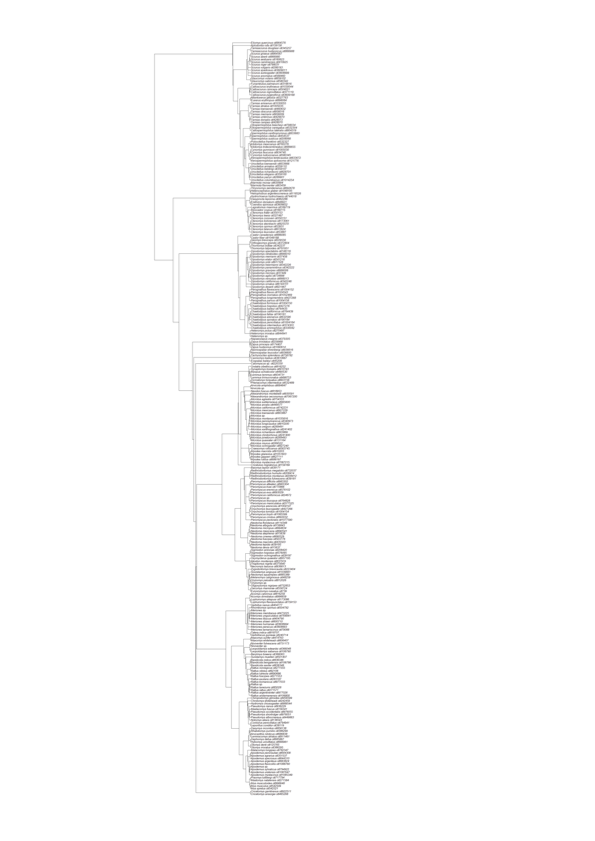

Supplement: Hunter-Barnett and Viney supplementary material 1 — Hunter-Barnett and Viney supplementary material [file S0031182024000556sup001.tiff]
