# Supplementary material for: Gut protozoa of wild rodents – a meta-analysis
Source: Parasitology. 2024 May 8;151(6):594–605. doi: 10.1017/S0031182024000556 (PMC11427965; doi:10.1017/S0031182024000556)
Supplement: Hunter-Barnett and Viney supplementary material 2 — Hunter-Barnett and Viney supplementary material [file S0031182024000556sup002.docx]

**Supplementary Table 1.** The digital object identifiers (DOIs) of the 344 articles identified in the methodical literature search. Articles are listed by publication year. If the DOI was not available for an article, the PubMed unique identifier (PMID) or JSTOR accession number is provided instead. If all three are not available, the article reference is given.

| **DOI** | **Year** | **DOI** | **Year** |
| --- | --- | --- | --- |
| 10.1016/j.ijppaw.2020.01.008 | 2020 | 10.1007/s00436-019-06530-4 | 2020 |
| 10.1186/s13071-019-3763-6 | 2019 | 10.1093/icb/icz136 | 2019 |
| 10.1016/j.ijppaw.2019.07.004 | 2019 | 10.1016/j.ijppaw.2019.03.017 | 2019 |
| 10.1016/j.heliyon.2019.e02382 | 2019 | 10.1016/j.ejop.2019.02.005 | 2019 |
| 10.1007/s00436-019-06502-8 | 2019 | PMID: 29922616 | 2018 |
| 10.3347/kjp.2018.56.1.93 | 2018 | 10.25225/fozo.v67.i2.a1.2018 | 2018 |
| 10.14202/vetworld.2018.293-296 | 2018 | 10.1186/s13071-018-3106-z | 2018 |
| 10.1186/s13071-018-2892-7 | 2018 | 10.1186/s13071-018-2802-z | 2018 |
| 10.1017/S0031182018001142 | 2018 | 10.1016/j.ympev.2018.05.009 | 2018 |
| 10.1016/j.ijppaw.2018.01.004 | 2018 | 10.1016/j.ijpara.2018.04.003 | 2018 |
| 10.1016/j.ejop.2018.02.001 | 2018 | 10.1016/j.ejop.2017.12.006 | 2018 |
| 10.1007/s11230-018-9788-y | 2018 | 10.1007/s11230-017-9771-z | 2018 |
| 10.1007/s00436-018-5973-9 | 2018 | 10.1007/s00436-018-5827-5 | 2018 |
| PMID: 28979348 | 2017 | PMID: 28935004 | 2017 |
| 10.1017/S0031182017001524 | 2017 | 10.1016/j.vetpar.2017.04.007 | 2017 |
| 10.1016/j.ijppaw.2017.05.003 | 2017 | 10.1016/j.ejop.2017.09.007 | 2017 |
| 10.1016/j.actatropica.2017.04.013 | 2017 | Rodríguez-Durán et al. 2015. Zootecnia Trop. 33(3):261-268 | 2016 |
| PMID: 28127340 | 2016 | PMID: 27244955 | 2016 |
| 10.7589/2015-01-010 | 2016 | 10.1894/0038-4909-61.4.331 | 2016 |
| 10.1654/1525-2647-83.1.122 | 2016 | 10.1638/2015-0055.1 | 2016 |
| 10.1371/journal.pone.0147090 | 2016 | 10.1186/s13071-016-1607-1 | 2016 |
| 10.1155/2016/6834206 | 2016 | 10.1155/2016/3263868 | 2016 |
| 10.1111/jeu.12249 | 2016 | 10.1016/j.vetpar.2015.10.017 | 2016 |
| 10.1016/j.parint.2016.03.010 | 2016 | 10.1016/j.meegid.2016.07.014 | 2016 |
| 10.1016/j.ejop.2016.04.008 | 2016 | 10.1007/s12639-015-0720-y | 2016 |
| 10.1002/jez.2003 | 2016 | PMID: 26114139 | 2015 |
| 10.7589/2014-04-099 | 2015 | 10.5604/12321966.1141359 | 2015 |
| 10.3347/kjp.2015.53.6.737 | 2015 | 10.3201/eid2112.141711 | 2015 |
| 10.1654/4689.1 | 2015 | 10.1080/00222933.2013.825025 | 2015 |
| 10.1017/S0031182014001929 | 2015 | 10.1016/j.meegid.2015.10.002 | 2015 |
| 10.1016/j.meegid.2015.03.003 | 2015 | 10.1016/j.ijppaw.2015.02.004 | 2015 |
| PMID: 25776597 | 2014 | PMID: 25642271 | 2014 |
| 10.2478/s11686-014-0304-5 | 2014 | 10.2478/bvip-2014-0033 | 2014 |
| 10.1890/13-2381.1 | 2014 | 10.1080/00222933.2013.867376 | 2014 |
| 10.1017/S003118201300139X | 2014 | 10.1016/j.meegid.2013.07.020 | 2014 |
| 10.1007/s11230-013-9466-z | 2014 | 10.7589/2013-02-028 | 2013 |
| 10.1645/GE-3144.1 | 2013 | 10.1645/12-94.1 | 2013 |
| 10.12816/0006382 | 2013 | 10.1186/1746-6148-9-229 | 2013 |
| 10.1128/AEM.01503-13 | 2013 | 10.1111/jzo.12076 | 2013 |
| 10.1098/rspb.2013.0598 | 2013 | 10.1098/rsbl.2013.0205 | 2013 |
| 10.1017/S0950268812002609 | 2013 | 10.1016/j.vetpar.2013.02.011 | 2013 |
| 10.1016/j.meegid.2013.01.011 | 2013 | 10.1016/j.ijpara.2013.04.007 | 2013 |
| 10.1016/j.exppara.2013.09.003 | 2013 | Kozerski et al. 2012. Arq. Ciênc. Vet. Zool. UNIPAR, Umuarama. 15(2):133-136. | 2012 |
| 10.5897/ajmr11.1356 | 2012 | 10.5812/jjm.3580 | 2012 |
| 10.2478/s11687-012-0033-y | 2012 | 10.2478/s11686-012-0016-7 | 2012 |
| 10.2478/s11686-012-0006-9 | 2012 | 10.1590/S1519-69842012000300019 | 2012 |
| 10.1139/Z2012-083 | 2012 | 10.1016/j.exppara.2012.05.009 | 2012 |
| 10.1007/s10344-011-0584-0 | 2012 | 10.1007/s00508-012-0237-7 | 2012 |
| 10.2478/s11687-011-0025-3 | 2011 | 10.1645/GE-2535.1 | 2011 |
| 10.1017/S0031182011001107 | 2011 | Futagbi et al. 2010. West Afr. J. Appl. Ecol. 17(1):81-87. | 2010 |
| 10.9775/kvfd.2009.1269 | 2010 | 10.7589/0090-3558-46.1.146 | 2010 |
| 10.1501/Vetfak_0000002378 | 2010 | 10.1501/Vetfak_0000002326 | 2010 |
| 10.1128/AEM.00687-10 | 2010 | 10.1111/j.1469-7998.2010.00734.x | 2010 |
| 10.1017/S003118200999179X | 2010 | 10.1016/j.vetpar.2010.02.012 | 2010 |
| Ozmen et al. 2009. Turkiye Parazitol. Derg. 33(3):245-247. | 2009 | PMID: 19887025 | 2009 |
| 10.2307/26327900 | 2009 | 10.1645/GE-1653.1 | 2009 |
| 10.1590/S0103-84782009005000085 | 2009 | 10.1128/AEM.01386-09 | 2009 |
| 10.1111/j.1365-2028.2008.01020.x | 2009 | 10.1590/S0103-84782008000200043 | 2008 |
| 10.14411/fp.2008.012 | 2008 | 10.1111/j.1749-4877.2008.00069.x | 2008 |
| 10.1017/S0031182008004952 | 2008 | 10.1007/s11230-008-9150-x | 2008 |
| PMID: 18247463 | 2007 | 10.1654/4269.1 | 2007 |
| 10.1590/S0074-02762007005000008 | 2007 | 10.1128/AEM.01034-07 | 2007 |
| 10.1128/AEM.00848-07 | 2007 | 10.1017/S0031182006001120 | 2007 |
| 10.1016/j.vetpar.2007.03.034 | 2007 | 10.1016/j.vetpar.2007.03.024 | 2007 |
| 10.1016/j.exppara.2007.03.014 | 2007 | 10.1007/s00436-007-0488-9 | 2007 |
| 10.1007/s00436-006-0276-y | 2007 | 10.1007/s00436-006-0251-7 | 2007 |
| 10.1002/hyp.6278 | 2007 | 10.1638/06-013.1 | 2006 |
| 10.1139/Z06-183 | 2006 | 10.1111/j.1600-0587.1998.tb00667.x | 2006 |
| 10.1111/j.1469-7998.2006.00089.x | 2006 | 10.7589/0090-3558-41.2.442 | 2005 |
| 10.1645/ge-564r.1 | 2005 | 10.1645/ge-466r.1 | 2005 |
| 10.1645/ge-3468.1 | 2005 | 10.1128/AEM.71.10.5929-5934.2005 | 2005 |
| 10.1051/parasite/2005124305 | 2005 | 10.1007/s00436-005-1459-7 | 2005 |
| 10.1007/s00436-005-1353-3 | 2005 | Drózdz et al. 2004. Helminthologia. 41(2):99-101. | 2004 |
| 10.1590/S0074-02762004000600008 | 2004 | 10.1292/jvms.66.983 | 2004 |
| 10.1128/AEM.70.12.7574-7577.2004 | 2004 | 10.1128/AEM.70.11.6748-6752.2004 | 2004 |
| 10.1016/j.vetpar.2004.04.005 | 2004 | 10.7589/0090-3558-39.4.762 | 2003 |
| 10.3201/eid0911.030084 | 2003 | 10.1292/jvms.65.1151 | 2003 |
| 10.1007/s00442-003-1345-x | 2003 | McAllister and Kessler 2002. J. Ark. Acad. Sci. 56(35):235-238 | 2002 |
| 10.5070/v420110115 | 2002 | 10.2307/3285505 | 2002 |
| 10.1654/1525-2647(2002)069[0211:ELAEOF]2.0.CO;2 | 2002 | 10.1139/w02-047 | 2002 |
| 10.1079/JOH2001109 | 2002 | 10.1017/S0031182002001865 | 2002 |
| 10.1016/S0304-4017(02)00181-4 | 2002 | 10.2307/3285195 | 2001 |
| 10.2307/3285191 | 2001 | 10.2307/3285044 | 2001 |
| 10.1674/0003-0031(2001)145[0409:POEAEI]2.0.CO;2 | 2001 | 10.1186/1751-0147-42-479 | 2001 |
| 10.1128/AEM.67.6.2840-2843.2001 | 2001 | 10.1128/AEM.67.3.1154-1162.2001 | 2001 |
| 10.1080/15627020.2001.11657112 | 2001 | 10.1017/S0952836901000504 | 2001 |
| 10.1017/S0031182001007259 | 2001 | 10.14411/fp.2000.020 | 2000 |
| 10.1017/S0031182099005545 | 2000 | 10.1016/S0932-4739(00)80037-1 | 2000 |
| 10.1016/s0932-4739(00)80023-1 | 2000 | 10.1016/S0304-4017(00)00331-9 | 2000 |
| O'Callaghan and O'Donoghue 1999. Trans. R. Soc. S. Aust. 123(4):133-135. | 1999 | 10.7589/0090-3558-35.4.660 | 1999 |
| 10.4067/S0716-07201999000300006 | 1999 | 10.2307/3285824 | 1999 |
| 10.1016/S0304-4017(98)00226-X | 1999 | Bomfim and Lopes 1998. Rev. Bras. Parasitol. Vet. 7(2):129-136. | 1998 |
| 10.15517/rbt.v46i2.19534 | 1998 | 10.2307/3284675 | 1998 |
| 10.2307/3284485 | 1998 | 10.1080/09603129873660 | 1998 |
| 10.1017/S0952836998001137 | 1998 | Bajer et al. 1997. Acta Parasitol. 42(4):192-194. | 1997 |
| 10.2307/3284455 | 1997 | 10.2307/3284412 | 1997 |
| 10.2307/3284341 | 1997 | 10.2307/2426760 | 1997 |
| 10.1051/parasite/1997044369 | 1997 | 10.1016/S0022-1759(96)00239-6 | 1997 |
| 10.1007/s004360050283 | 1997 | 10.2307/3545876 | 1996 |
| 10.2307/3284150 | 1996 | 10.2307/2265712 | 1996 |
| 10.1016/S0934-8840(96)80106-X | 1996 | 10.1016/S0035-9203(96)90235-7 | 1996 |
| McKnown et al. 1995. J. Helminthol. Soc. Wash. 62(1):89-93. | 1995 | 10.5962/bhl.title.156442 | 1995 |
| 10.2307/3283946 | 1995 | 10.2307/3283928 | 1995 |
| 10.1590/S0074-02761995000600002 | 1995 | 10.1017/S0031182000081804 | 1995 |
| Thomas and Stanton 1994. J. Helminthol. Soc. Wash. 61(1):17-21. | 1994 | 10.7589/0090-3558-30.3.450 | 1994 |
| 10.7589/0090-3558-30.3.359 | 1994 | 10.7589/0090-3558-30.1.110 | 1994 |
| 10.2307/3283755 | 1994 | 10.1139/z94-032 | 1994 |
| 10.1016/S0932-4739(11)80025-8 | 1994 | PMID: 8013931 | 1993 |
| McAllister et al. 1993. J. Helminthol. Soc. Wash. 60(2):256-258. | 1993 | 10.7589/0090-3558-29.1.161 | 1993 |
| 10.2307/3283744 | 1993 | 10.2307/3283743 | 1993 |
| 10.2307/3283505 | 1993 | 10.1080/09603129309356785 | 1993 |
| JSTOR: 41712736 | 1992 | 10.2307/3283636 | 1992 |
| 10.2307/3283482 | 1992 | 10.2307/3283322 | 1992 |
| 10.1111/j.1550-7408.1992.tb04866.x | 1992 | 10.1080/00480169.1992.35693 | 1992 |
| PMID: 1937279 | 1991 | 10.2307/3282760 | 1991 |
| 10.2307/3226742 | 1991 | 10.1016/S0003-9365(11)80187-9 | 1991 |
| Miyaji et al. 1989. Jpn. J. Parasitol. 38:368-372. | 1990 | Yamaura et al. 1990. Jpn. J. Parasitol. 39:439-444. | 1990 |
| JSTOR: 41712612 | 1990 | 10.2307/3282798 | 1990 |
| 10.2307/3282659 | 1990 | 10.1128/aem.56.1.31-36.1990 | 1990 |
| 10.1111/j.1550-7408.1990.tb01261.x | 1990 | 10.1111/j.1550-7408.1990.tb01115.x | 1990 |
| Seville and Williams 1989. Proc. Helminthol. Soc. Wash. 56(2):204-206. | 1989 | 10.2307/3282919 | 1989 |
| 10.1111/j.1550-7408.1989.tb01068.x | 1989 | 10.1017/S0031182000061394 | 1989 |
| 10.2508/chikusan.59.351 | 1988 | 10.2307/3282497 | 1988 |
| 10.2307/3282226 | 1988 | 10.2307/3226510 | 1988 |
| 10.1258/002367788780746665 | 1988 | 10.1128/aem.54.11.2777-2785.1988 | 1988 |
| JSTOR: 44539406 | 1987 | 10.2307/3282103 | 1987 |
| Kietzmann and Kietzmann 1987. Proc. Iowa Acad. Sci. 94(1):22-23. | 1987 | 10.7589/0090-3558-23.4.576 | 1987 |
| 10.1128/aem.53.8.1790-1792.1987 | 1987 | 10.1128/aem.53.7.1574-1579.1987 | 1987 |
| 10.7589/0090-3558-22.1.115 | 1986 | 10.1139/m86-171 | 1986 |
| 10.1111/j.1550-7408.1986.tb05607.x | 1986 | Upton et al. 1985. Proc. Helminthol. Soc. Wash. 55(1):60-63. | 1985 |
| 10.2307/3282011 | 1985 | 10.2307/3281719 | 1985 |
| 10.2307/3281445 | 1985 | 10.2307/3281432 | 1985 |
| 10.1128/aem.50.1.177-178.1985 | 1985 | 10.1111/j.1550-7408.1985.tb04059.x | 1985 |
| 10.7589/0090-3558-20.4.279 | 1984 | 10.2307/3281661 | 1984 |
| 10.1111/j.1550-7408.1984.tb02976.x | 1984 | 10.1111/j.1469-7998.1984.tb05089.x | 1984 |
| 10.2307/3281301 | 1983 | 10.1111/j.1365-2907.1983.tb00276.x | 1983 |
| 10.1007/BF00009160 | 1983 | 10.2307/3281108 | 1982 |
| 10.1111/j.1550-7408.1982.tb04006.x | 1982 | 10.1017/S0031182000056225 | 1982 |
| JSTOR: 44536562 | 1980 | 10.2307/3280827 | 1980 |
| 10.1111/j.1550-7408.1980.tb05376.x | 1980 | 10.1093/oxfordjournals.aje.a113019 | 1980 |
| Ryšavý and Černá 1979. Folia Parasitol. 26(1):65-68. | 1979 | Fleming et al. 1979. Proc. Helminthol. Soc. Wash. 46(1):115-127. | 1979 |
| Levine and Husar 1979. Proc. Helminthol. Soc. Wash. 46(1):135-137. | 1979 | 10.1111/j.1550-7408.1979.tb04189.x | 1979 |
| 10.2307/3279841 | 1977 | 10.1111/j.1550-7408.1977.tb00966.x | 1977 |
| 10.1258/002367776781035314 | 1976 | 10.2307/3278353 | 1974 |
| 10.1111/j.1550-7408.1972.tb03453.x | 1972 | 10.1111/j.1550-7408.1972.tb03429.x | 1972 |
| 10.2307/3278801 | 1971 | 10.2307/3277983 | 1971 |
| 10.2307/1378687 | 1971 | 10.1139/z71-109 | 1971 |
| 10.1111/j.1550-7408.1971.tb03383.x | 1971 | 10.1111/j.1550-7408.1971.tb03379.x | 1971 |
| 10.1111/j.1550-7408.1971.tb03311.x | 1971 | de Vos and Dobson 1970. Onderstepoort J. Vet. Res. 37(4):185-190. | 1970 |
| 10.7589/0090-3558-6.2.107 | 1970 | 10.2307/3277446 | 1970 |
| 10.1139/z70-109 | 1970 | 10.1111/j.1550-7408.1980.tb05377.x | 1970 |
| 10.1111/j.1550-7408.1970.tb02368.x | 1970 | 10.1139/z69-123 | 1969 |
| 10.1111/j.1550-7408.1970.tb04713.x | 1969 | 10.1016/0014-4894(69)90107-6 | 1969 |
| 10.2307/3277107 | 1968 | 10.1111/j.1550-7408.1968.tb02203.x | 1968 |
| 10.1111/j.1550-7408.1968.tb02152.x | 1968 | 10.1111/j.1550-7408.1968.tb02129.x | 1968 |
| 10.1111/j.1550-7408.1968.tb02121.x | 1968 | 10.1111/j.1550-7408.1968.tb02083.x | 1968 |
| 10.1080/00034983.1968.11686557 | 1968 | 10.1111/j.1550-7408.1967.tb02044.x | 1967 |
| 10.1111/j.1550-7408.1967.tb02040.x | 1967 | 10.1111/j.1550-7408.1967.tb01980.x | 1967 |
| 10.1111/j.1550-7408.1966.tb01961.x | 1966 | 10.1111/j.1550-7408.1965.tb03245.x | 1965 |
| 10.1111/j.1550-7408.1965.tb03236.x | 1965 | 10.1111/j.1550-7408.1964.tb01724.x | 1964 |
| 10.2307/3275782 | 1963 | 10.1111/j.1550-7408.1962.tb02615.x | 1962 |
| 10.2307/3275485 | 1961 | 10.2307/3274986 | 1961 |
| 10.2307/3275173 | 1960 | 10.1111/j.1550-7408.1960.tb00731.x | 1960 |
| 10.1111/j.1550-7408.1960.tb00721.x | 1960 | 10.1111/j.1550-7408.1960.tb00709.x | 1960 |
| 10.1080/00034983.1960.11685992 | 1960 | 10.1111/j.1550-7408.1959.tb04360.x | 1959 |
| 10.1111/j.1469-7998.1959.tb05528.x | 1959 | 10.1111/j.1550-7408.1958.tb02530.x | 1958 |
| 10.1080/00034983.1958.11685840 | 1958 | Levine et al. 1957. Trans. Ill. Acad. Sci. 50:291-299. | 1957 |
| 10.1111/j.1550-7408.1957.tb02491.x | 1957 | 10.1111/j.1096-3642.1957.tb00272.x | 1957 |
| 10.2307/3274144 | 1955 | 10.1017/S0022172400036123 | 1948 |
| 10.1017/S0031182000010763 | 1938 | 10.1093/oxfordjournals.aje.a118249 | 1936 |
| 10.1017/S0031182000023866 | 1934 | 10.1093/oxfordjournals.aje.a117882 | 1932 |
| 10.1111/j.1096-3642.1931.tb01037.x | 1931 | 10.1001/jama.1915.02580260026009 | 1915 |
