# Supplementary material for: Gut protozoa of wild rodents – a meta-analysis
Source: Parasitology. 2024 May 8;151(6):594–605. doi: 10.1017/S0031182024000556 (PMC11427965; doi:10.1017/S0031182024000556)
Supplement: Hunter-Barnett and Viney supplementary material 5 — Hunter-Barnett and Viney supplementary material [file S0031182024000556sup005.docx]

Supplementary Table 4. The average gut protozoa prevalence for each rodent species.

Protozoa richness is the number of protozoa genera found within the gut of that host species.

| **Species** | **Protozoa richness** | **Prevalence (%)** | **Sample size (k)** | **95 % CI** | |
| --- | --- | --- | --- | --- | --- |
|  |  |  |  | **Lower** | **Upper** |
| *Apodemus agrarius* | 3 | 25.8 | 29 | 11.8 | 42.0 |
| *Apodemus flavicollis* | 3 | 23.3 | 54 | 14.9 | 32.6 |
| *Apodemus speciosus* | 4 | 33.1 | 12 | 3.1 | 71.8 |
| *Apodemus sylvaticus* | 7 | 23.2 | 43 | 13.5 | 34.2 |
| *Castor canadensis* | 3 | 6.3 | 27 | 2.1 | 11.8 |
| *Dipodomys merriami* | 1 | 14.1 | 11 | 1.4 | 33.0 |
| *Dipodomys ordii* | 1 | 5.2 | 10 | 0.0 | 26.7 |
| *Microtus agrestis* | 8 | 43.4 | 23 | 21.0 | 67.0 |
| *Microtus arvalis* | 4 | 42.5 | 22 | 10.3 | 77.9 |
| *Microtus* | 5 | 36.2 | 10 | 14.3 | 60.8 |
| *Mus musculus* | 8 | 7.1 | 68 | 3.0 | 12.4 |
| *Myodes glareolus* | 8 | 32.5 | 51 | 22.1 | 43.6 |
| *Ondatra zibethicus* | 7 | 47.8 | 26 | 31.5 | 64.3 |
| *Peromyscus* | 6 | 5.9 | 19 | 0.0 | 19.2 |
| *Rattus norvegicus* | 13 | 9.1 | 55 | 3.3 | 16.7 |
| *Rattus rattus* | 11 | 23.6 | 41 | 14.0 | 34.4 |
| *Sciurus carolinensis* | 3 | 70.4 | 15 | 26.6 | 99.9 |
| *Sciurus vulgaris* | 3 | 53.8 | 10 | 25.6 | 81.0 |
| *Sigmodon hispidus* | 5 | 13.0 | 12 | 5.6 | 22.1 |
